# Evaluating cochlear implant outcomes in DFNA9 subjects: a comprehensive study on cerebral white matter lesions and vestibular abnormalities

**DOI:** 10.1007/s00405-024-08933-1

**Published:** 2024-09-13

**Authors:** M.L.A. Fehrmann, F.J.A. Meijer, E.A.M. Mylanus, R.J.E. Pennings, C.P. Lanting, W.J. Huinck

**Affiliations:** 1 Department of Otorhinolaryngology, Radboud University Medical Center, Nijmegen, The Netherlands; 2 Donders Institute for Brain, Cognition and Behaviour, Nijmegen, The Netherlands; 3 Department of Medical Imaging, Radboud University Medical Center, Nijmegen, The Netherlands

**Keywords:** DFNA9, Cochlear implant outcomes, MRI, White matter lesions, Fazekas score, Semicircular canals

## Abstract

**Purpose:**

This study assessed whether the Fazekas score could account for the variability in cochlear implantation (CI) outcomes among individuals with DFNA9 and evaluated signal loss in the semicircular canals (SCCs) on magnetic resonance imaging (MRI) among individuals with DFNA9.

**Method:**

This retrospective cross-sectional study included CI recipients with DFNA9. Pre-implantation MRI-scans were reviewed to determine the Fazekas score, localizing and grading cerebral white matter lesions (WML), and identify abnormalities in the SCCs. CI performance was assessed by evaluating phoneme scores one year post-implantation. The function of the SCCs was evaluated using rotatory chair testing with electronystagmography (ENG) and the video Head Impulse Test (vHIT).

**Results:**

Forty-five subjects (49 ears) were enrolled. The phoneme scores significantly improved from 35% (IQR 11–50) pre-implantation to 84% (IQR 76–90) one year post-implantation. No correlation was observed between the Fazekas score and the one-year post-implantation phoneme score (r_sp_=0.003, *p* = 0.986). Signal loss in at least one SCCs was detected in 97.7% of subjects and 77.8% of ears. There was no correlation between vestibular test results and fluid signal loss in the SCCs on MRI.

**Conclusion:**

Most individuals with DFNA9 show improved speech recognition with CI. The observed variability in CI outcomes was not linked to the Fazekas score. Additionally, our study confirms a high prevalence of focal sclerosis in DFNA9. Recognizing the limitations of this study, further research is needed to explore the predictive role of the Fazekas score on CI outcomes and its relationship with vestibular function.

**Supplementary Information:**

The online version contains supplementary material available at 10.1007/s00405-024-08933-1.

## Introduction

DFNA9 is a progressive type of non-syndromic sensorineural hearing loss (SNHL) inherited in an autosomal dominant manner, typically emerging in mid-life due to mono-allelic pathogenic variants in *COCH* [1]. It often includes vestibular deterioration, emerging between the third and seventh decade, depending on the genotype. Self-reported onset ages for SNHL range from the second to seventh decade, with SNHL typically beginning around the fourth decade in the most common p.Pro51Ser variant [1].

Hearing rehabilitation in individuals with DFNA9 often starts with hearing aids (HA), but many eventually require a cochlear implant (CI) to achieve adequate speech understanding due to its progressive nature. Vermeire et al. evaluated CI outcomes in DFNA9, showing significant improvement in speech recognition and quality of life post-implantation, although with noticeable variability in speech recognition scores [2].

This variability in CI outcomes has been attributed to multiple subject-specific factors [3–6]. Lazard et al. identified nine factors in a model explaining 22% of CI outcome variance, with duration of severe/profound SNHL, age of onset SNHL, duration of CI experience, and aetiology having the strongest impact [4, 5]. In addition to these factors, cognitive factors are increasingly recognized for their impact on CI outcomes [6–10], as accurate sound perception and cognitive skills are both essential for accurate sentence recognition [9]. These cognitive skills rely on various cerebral grey matter regions, requiring effective information transfer between these regions, facilitated by white matter [11]. Consequently, cerebral white matter lesions (WML) can disrupt central processing and affect speech understanding [12–15]. WML are frequently related to small vessel disease, associated with vascular risk factors and commonly observed in the ageing population [16]. Previous studies linked cerebral WML to lower speech recognition in normal hearing individuals under 70 years of age [15], promoting investigation into their role in CI outcomes [6].

Optimal visualization of WML involves identifying hyperintensities on T2-weighted and fluid-attenuated inversion recovery (FLAIR) sequences on brain magnetic resonance imaging (MRI). The Fazekas scoring system, distinguishing periventricular (PVWM) and deep white matter lesions (DWM; Table 1), is commonly used to localize and grade WML [17]. Knopke et al. explored the correlation between Fazekas score and speech recognition with CI in a heterogenous study cohort, finding that the PVWM score accounted for 27.4% of speech perception variance in subjects aged 50–70 [6]. Our study aims to investigate this correlation in a more homogenous cohort, including DFNA9 subjects, who typically receive CI in the same age range (50–70) and have normal cochleovestibular anatomy [1]. Therefore, this study’s primary objective is to assess the correlation between CI outcomes and WML localized and graded with the Fazekas score in DFNA9 CI recipients.

**Table 1:** The Fazekas scoring system

**Periventricular white matter lesions (PVWM)**	
0	Absent
1	Caps or pencil-thin lining
2	Smooth halo
3	Irregular periventricular signal extending into the deep white matter
**Deep white matter lesions (DWM)**	
0	Absent
1	Punctate foci
2	Beginning confluence
3	Large confluent areas

The Fazekas total score is a sum of the PVWM score and the DWM score

Besides SNHL, individuals with DFNA9 also exhibit vestibular deterioration, presenting with vertigo and balance problems [1]. Studies have documented the presence of focal sclerosis and narrowing in at least one semicircular canal (SCC) on computer tomography (CT) or signal loss on T2-weighted MRI in DFNA9 individuals [18–20], consistent with histopathology findings, showing new bone and fibrous tissue formation within SCCs [21]. These studies consistently highlight the higher sensitivity of MRI in detecting these radiologic abnormalities compared to CT [18–20]. It is proposed that this phenomenon can be attributed to the pathophysiology of DFNA9, wherein fibrosis, for which initially MRI is the most sensitive modality, is followed by calcification or advanced fibrosis, detectable by CT as well [19, 20]. These studies suggest that imaging abnormalities may serve as biomarkers for vestibular dysfunction in DFNA9 [18–20]. To provide additional evidence, this study’s second aim is to evaluate the signal loss in the SCCs on MRI in Dutch DFNA9 individuals.

## Methods

### Study design and population

This is a retrospective, observational, cohort study including subjects with DFNA9. Subjects were enrolled if they met the following criteria: (1) They had a clinical diagnosis of DFNA9 confirmed by a genetic diagnosis identifying one monoallelic pathogenic variant in *COCH* or exhibited the typical phenotype along with the identification of a pathogenic variant in *COCH* in a sibling; (2) The speech recognition scores were evaluated at one-year post-implantation; and (3) A pre-implantation MRI scan was available. Subjects were excluded if they had a condition that might affect CI performance or when the MRI scan was performed ≥ 4 years pre-implantation, considering the considerable advancement of WML in the elderly within five years [22]. As a result, the time between the MRI and the one-year follow-up is ≤ 5 years.

### Data collection

#### Demographic data

Demographic data were obtained through a review of medical records. They included gender, self-reported age of onset of SNHL, use of HA before implantation, and age at the time of implantation. The genetic diagnosis was gathered by scoring the variant(s) with the associated protein change(s). No additional genetic analyses were performed. Furthermore, data regarding cardiovascular risk factors were gathered, including hypertension, diabetes mellitus (DM), elevated cholesterol levels, current or former smoking habits, the use of anticoagulant drugs, and a history of myocardial infarction and/or stroke. Additionally, the American Society of Anaesthesiologists (ASA) score was obtained, ranging from one to six. Anaesthesiologists assign this score during preoperative screening as a subjective assessment of the subject’s overall health [23].

#### Audiological performance

Audiometry data obtained through reviewing medical records was evaluated. Hearing assessments were conducted using standard pure tone and speech audiometry in accordance with current local protocols. No additional auditory tests were performed. The pure tone average (PTA) was determined using thresholds at 500, 1000, 2000, and 4000 Hz (PTA_0.5–4 kHz_). Phoneme scores were assessed in quiet at 65 dB SPL. For both PTA and phoneme scores, aided and unaided scores were measured pre-implantation, while only aided scores were measured post-implantation.

Not all subjects used a HA in the ear to be implanted. We calculated the best-aided PTA and phoneme score to represent the pre-implantation auditory performance. For the best-aided scores, we recorded the aided scores using HA in the ear to be implanted or the unaided score when subjects did not use a HA in the ear to be implanted. These best-aided scores were used to compare pre-implantation hearing performance with post-implantation CI performance. The post-implantation PTA_0.5–4 kHz_ and phoneme scores at 65 dB SPL were evaluated at one year post-implantation.

#### Radiological assessment

Pre-implantation MRI scans were retrospectively reviewed by an experienced neuroradiologist. The scanning protocol of the brain MRIs was not standardized but included at least an adequate T2 sequence. The WMLs were graded according to the Fazekas classification system, which distinguishes PVWM and DWM lesions (Table 1) [17]. The Fazekas score is the sum of the PVWM and DWM scores and ranges from zero to six. Additionally, the pre-implantation MRI scans were reviewed to identify abnormalities at the level of the SCCs. Narrowing or signal loss of each SCCs of both sides were scored for each subject. Any additional brain or cochleovestibular abnormalities (including cerebral infarctions) were also reported when applicable.

#### Vestibular function

Vestibular data was obtained by reviewing medical records. When tested, a comprehensive assessment of semi-circular canal function involved rotatory chair testing using electronystagmography (ENG) and the video Head Impulse Test (vHIT) to assess the individual canals. In some cases, subjects had a clear diagnosis of DFNA9 based on their phenotype, family history, and/or genetic testing. In these cases, it was presumed that subjects exhibited areflexia, so vestibular tests were not conducted.

### Data analysis

Statistical analyses were conducted using IBM Statistical Package for the Social Sciences (SPSS) version 29. A p-value of < 0.05 was considered statistically significant. Figures were created with Prism version 10. To assess whether the data deviated from a normal distribution, the Shapiro-Wilk test was performed. Normally distributed data were presented as mean with standard deviation (e.g., age during implantation, self-reported duration of SNHL, and months/years of follow-up). In contrast, non-normally distributed data were expressed as median with interquartile ranges (IQR, e.g., PTA-scores, phoneme scores, and Fazekas score). Spearman’s rank coefficient (r_sp_) and univariate linear regressions analysis were conducted to assess the correlation between one-year post-implantation phoneme scores and categorical variables, including the Fazekas score and additional brain abnormalities on MRI (e.g., cerebellar infarction, lacunar infarction). Pearson’s correlation was utilized to examine the correlation between one-year post-implantation phoneme scores and continuous variables, including age during implantation, self-reported duration of SNHL, and duration of HA use in the ear to be implanted pre-implantation, and degree of SNHL pre-implantation was examined using the same tests. Multiple regression analysis was used to evaluate the impacts of these variables on the one-year post-implantation phoneme scores.

Since Schmidt et al.’s review showed that progression of WML could also be observed even after a two-year follow-up [24], we conducted a separate analysis to examine the correlation between the Fazekas score and the phoneme score in subjects with a duration of ≤ 24 months between the MRI and implantation. Spearman’s rank correlation coefficient (r_sp_) was utilized to evaluate the correlation between MRI findings and vestibular results.

## Results

### Subjects

Following the in- and exclusion criteria assessment, 45 subjects with DFNA9 were enrolled in this study (Table 2, and Supplementary Table 1). Pathogenic variants in *COCH* were identified in 30 subjects, in which the common p.Pro51Ser variant was predominantly found. A total of 49 cochlear implantations were performed at a mean age of 65 ± 5.9 years (Table 2). Four subjects received bilateral implants as part of their participation in a research study. A HA was used prior to implantation in 43 ears (87.8%).

**Table 2:** Subject characteristics

Subject characteristic	*N* = 45 subjects (100%)	
**Gender, % female**	28	(62.2)
**Implantation**		
Unilateral	40	(88.9)
Bilateral simultaneously	3	(6.7)
Bilateral sequentially	2	(4.4)
**Variant in ** ***COCH***		
c.151 C > T (p.(Pro51Ser))	29	(64.4)
c.263G > A (p.(Gly88Glu))	1	(2.2)
Unknown*	15	(33.3)
**Pre-implantation ASA score**		
1	16	(32.0)
2	28	(56.0)
3	6	(12.0)
**Cardiovascular risk factors**		
None	26	(57.8)
Hypertension	13	(28.9)
Diabetes Mellitus	9	(20.0)
High cholesterol	2	(4.4)
Smoking	5	(11.1)
Anticoagulant drug use	6	(13.3)
History of myocardial infarction	3	(6.7)
History of stroke	1	(2.2)
Ear characteristic	***N*** ** = 49 ears (100%)**	
**Age at implantation**	65 ± 5.9 y	
**Self-reported duration of hearing loss prior to implantation**	18 ± 6.3 y	
Degree HL pre-implantation**		
Severe (61–80 dB HL)	9	(18.4)
Profound (> 80 dB HL)	40	(81.6)
**Hearing aid in ear to be implanted**	43	(87.8)

ASA indicates American Society of Anaesthesiologists; SD, standard deviation; HL, hearing loss; y, years

* The variant was unidentified, either because a detailed genetic test report was unavailable (*N* = 9) or because the variant was detected in a sibling (*N* = 6)

**According to WHO’s grades of hearing impairment

### Audiological outcomes

**Fig. 1:**
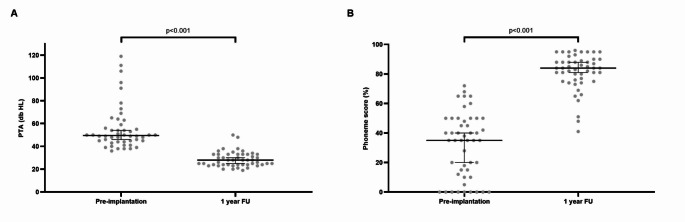
Cochlear implant outcomes. PTA indicates pure tone average; FU, follow-up. Pre-implantation scores represent the best-aided PTA_0,5–4 kHz_ and phoneme scores. **A**. Individual data with median and inter quartile ranges (IQR) of PTA_0,5–4 kHz_ scores of each ear. B. Boxplot of phoneme scores at 65 dB SPL in quiet of each ear. Pre-implantation aided phoneme scores were not available in all subjects

The median pre-operative unaided PTA_0,5–4 kHz_ was 94 dB HL (IQR 86–109), while the median pre-operative best-aided PTA_0,5–4 kHz_ was 50 dB HL (IQR 44–58). The latter significantly improved to a median of 28 dB HL (IQR 24–33) at 14.1 ± 2.3 months post-implantation (p = < 0.001; *N* = 48; Fig. 1A).

The median best-aided pre-operative phoneme score at 65 dB SPL in quiet was 35% (IQR 11–50; *N* = 49 ears). With a mean follow-up of 13.9 ± 2.4 months post-implantation, the median phoneme score significantly increased to 84% (IQR 76–90; *N* = 49 ears; p = < 0.001; Fig. 1B). Ears rehabilitated with HA pre-implantation (*N* = 43 ears) did not show significantly higher phoneme scores (*p* = 0.561) compared to ears without HA (*N* = 6 ears).

Seven subjects (15.6%) with poorer outcomes were identified (i.e., a phoneme score < 70% at 65 dB SPL one year post-implantation or non-use). Among them, four showed improvement over time, with phoneme scores rising to 71–90%. All three subjects who maintained phoneme scores < 70% reported a prolonged duration of SNHL ranging from 15 to 20 years, and two of them stopped using their HA pre-implantation due to a perceived lack of benefit. This indicates that all three subjects experienced a prolonged period without adequate auditory stimulation. One subject became a non-user, while the other two reported being satisfied with the outcomes despite the lower phoneme scores, noting improved communication abilities and social interactions.

### Cerebral MRI findings and the Fazekas score

**Fig. 2:**
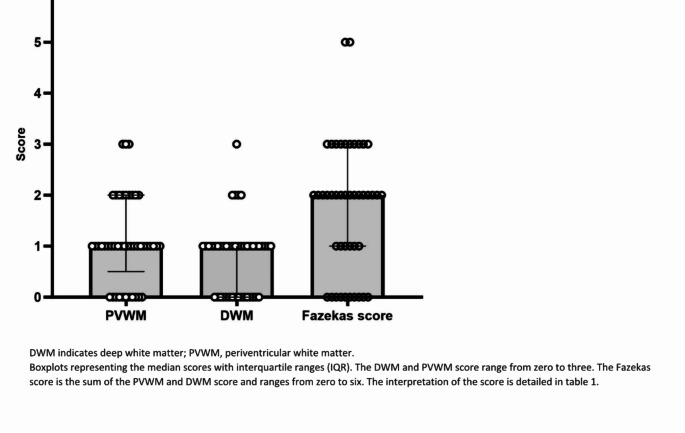
Fazekas score. DWM indicates deep white matter; PVWM, periventricular white matter. Boxplots representing the median scores with interquartile ranges (IQR). The DWM and PVWM score range from zero to three. The Fazekas score is the sum of the PVWM and DWM score and ranges from zero to six. The interpretation of the score is detailed in Table 1

In the total study population, the mean age at the MRI was 64 ± 5.6 years, and it was performed seven months (IQR 4–15) pre-implantation. In most subjects, a T2-weighted sequence was used (*N* = 41; 91.1%; Table 3). Cerebral WML were observed in 35 individuals and the median Fazekas score was 2 (IQR 1–3; Fig. 2). The median PVWM score was 1 (IQR 1–2), while the DWM score was 1 (IQR 0–1). Additional brain abnormalities were identified in ten subjects (Table 3).

**Table 3:** Magnetic Resonance Imaging (MRI) and vestibular findings

Subject characteristic	*N* = 45 subjects (100%)
**Age at time of MRI**	64 ± 5.6 y
**Time between MRI and implantation**	7 m (IQR 4–15)
**MRI sequence**	
T2-weighted	41 (91.1)
FLAIR	1 (2.2)
T2/FLAIR	3 (6.7)
**Fazekas score**	
PVWM	1 (IQR 1–2)
DWM	1 (IQR 0–1)
Fazekas	2 (IQR 1–3)
**Additional brain abnormalities**	
Previous lacunar infarction	4 (8.8)
Cerebellar infarction	1 (2.2)
Cerebellar and lacunar infarction	2 (4.4)
Infarct left frontal lobe	1 (2.2)
Tissue loss left frontal lobe	1 (2.2)
Tissue loss left parietal lobe	1 (2.2)
**SCC characteristics**	***N*** ** = 90 ears (100%)**
**Signal loss SCC**	
Any canal	70 (77.8)
Superior canal	44 (48.9)
Lateral canal	32 (35.6)
Posterior canal	31 (34.4)
Missing*	2 (2.2)
**ENG**	
Areflexia	68 (75.6)
Not performed	22 (24.2)
**vHIT**	
Areflexia superior canal	31 (34.4)
Areflexia lateral canal	31 (34.4)
Areflexia posterior canal	32 (35.6)
Not performed	58 (64.4)

MRI indicates magnetic resonance imaging; FLAIR, fluid-attenuated inversion recovery; PVWM, periventricular with matter; DWM, deep white matter; SCC, semicircular canal; ENG, electronystagmography; vHIT, video head impulse test; y, years; m, months; IQR, interquartile ranges

* Signal loss at the level of the SSC was not assessable at the MRI scan of one subject

No correlation was found between the Fazekas score and the phoneme score at 65 dB SPL in quiet one-year post-implantation (r_sp_=0.003; *p* = 0.986). Also, neither the PVWM score (r_sp_=0.022; *p* = 0.822) nor the DWM score (r_sp_=0.031; *p* = 0.835) was correlated with speech recognition one-year post-implantation (Supplementary Table 2). Additional brain abnormalities on MRI, including cerebellar infarction, lacunar infarction, left frontal lobe infarction, or tissue loss left frontal or parietal lobe, were also not correlated with the phoneme score at 65 dB SPL one-year post-implantation (r_sp_=0.086; *p* = 0.558). No other known factors influencing CI outcomes, including age at implantation, self-reported duration of SNHL, HA in the ear to be implanted pre-implantation, and degree of SNHL pre-implantation, were significantly correlated with speech recognition one year post-implantation (Supplementary Table 2). When incorporating these factors into a multiple regression model alongside the Fazekas score and additional brain abnormalities, it was observed that none of these variables significantly influenced speech recognition outcomes at one-year post-implantation (Table 4).

**Table 4:** Multivariable regression analysis with the phoneme scores at 65 dB SPL in quiet as dependent variable

Variable	Unstandardized B	Standardized coefficients Beta	Standard Error	*p*
Constant	64.069		22.754	0.007
Fazekas	-0.624	-0.071	1.573	0.694
Additional brain abnormalities on MRI	3.1644	0.104	4.841	0.517
Age during implantation	0.173	0.082	0.382	0.653
Self-reported duration of HL	0.351	0.178	0.331	0.295
Hearing aid in ear to be implanted	4.964	0.133	5.653	0.385
Degree of SNHL pre-implantation	-0.041	-0.047	0.143	0.776

P indicates significancy. R^2^ = 0.073, *p* = 0.768

To account for the potential progression of WML in individuals with > 24 months between the MRI and implantation, we conducted a separate analysis to examine the correlation between the Fazekas score and the phoneme score in subjects with a duration of ≤ 24 months between the MRI and implantation (*N* = 41 ears). Again, no correlation was observed between the Fazekas score and the phoneme score at 65 dB SPL in quiet one-year post-implantation (r_sp_=0.035; *p* = 0.819). Similarly, neither the PVWM score (r_sp_=0.059; *p* = 0.702) nor the DWM score (r_sp_=0.001; *p* = 0.997) correlated with speech recognition one year after implantation.

### Vestibular MRI findings and vestibular function

Signal loss at the level of the SCCs on T2-weighted MRI was assessable in 44 subjects (88 ears). In 43 out of 44 subjects (97.7%) and 70 out of 88 ears (77.8%), a signal loss in at least one of the SCCs was observed. The superior SCC was most frequently affected (48.9%; Table 3). Analyses were conducted to see if signal loss in the SCCs was linked to more advanced disease. Factors like longer self-reported SNHL duration, older age at MRI, and higher SNHL severity (higher unaided PTA_0,5–4 kHz_ pre-implantation) were included but showed no significant correlations.

Among the 44 subjects assessed for signal loss in the SCCs, all reported vestibular complaints, either current or in the past. There was, however, insufficient data to evaluate the self-reported age of onset of vestibular complaints. Within this group, vestibular function was tested in 35 subjects (79.5%). ENG was performed in 34 subjects, revealing bilateral areflexia in all cases. The vHIT was conducted in 16 subjects, with 15 exhibiting areflexia in all SCCs and one showing normal function in the right superior and lateral SCC. No correlation was found between vHIT results (normal or areflexia) and signal loss in the SCCs on MRI (r_sp_=0.116; *p* = 0.262).

## Discussion

This study demonstrated that cochlear implantation is a successful type of rehabilitation for DFNA9 subjects. The wide range in outcomes was not associated with cognitive performance measured by the degree and localisation of cerebral WML. Additionally, this study showed that nearly all individuals with DFNA9, who exhibit besides SNHL also vestibular areflexia, had signs of focal sclerosis in at least one of the SCCs on T2-weighted MRI.

### Cochlear implant outcomes in DFNA9

Limited literature exists on CI outcomes in individuals with DFNA9. Vermeire et al. studied eleven subjects, reporting significant improvement in speech recognition in quiet post-implantation and enhanced quality of life. Their performance varied widely, ranging from 20 to 90%, with a mean score of 64% [2]. In our study with 45 subjects (49 ears), the median phoneme score one-year post-implantation was 84% (range 41–96%), indicating overall favourable outcomes, although with a wide variability.

However, 6.7% of subjects had poorer outcomes (phoneme score < 70% of non-use post-implantation). These individuals experience prolonged periods of auditory deprivation (SNHL duration: 15–20 years), and discontinued HA use before implantation. These factors negatively impact CI outcomes in post-lingual adults [5, 25].

### Predictive value of the Fazekas score

Previous studies proposed a model incorporating nine factors explaining 22% of CI outcome variance, leaving much unexplained [5]. Recent research introduced additional factors like genetic aetiology [26], electrocochleography [10, 27], and the Fazekas score [6]. We assessed if the Fazekas score, grading and localizing cerebral WML, could explain CI outcome variability in DFNA9 individuals. However, we found no significant correlation between the total Fazekas score and phoneme scores in quiet one year post-implantation. Additionally, neither the PVWM nor the DWM score correlated with speech recognition scores one year post-implantation.

There are a couple of potential reasons why we did not find a correlation between the Fazekas score and CI outcomes. Firstly, it is plausible that we could not accurately ascertain the Fazekas score. FLAIR sequences are particularly effective in detecting WML by suppressing cerebrospinal fluid and emphasizing structural changes in white matter. Consequently, assessing WML without a FLAIR sequence is suboptimal. Given the retrospective design of this study, we re-assessed the available MRI scans, which were initially conducted to examine the anatomy of the inner ear and vestibular organ. These scans did, in general, not include FLAIR sequences because the focus was not on brain assessment. As FLAIR sequences were only available in four individuals, this might have led to an inaccurate Fazekas score.

Secondly, using phoneme scores in quiet, although also used in Knopke et al.’s study, may not be the most sensitive outcome measure to evaluate the correlation between the Fazekas score and CI performance. Cognitive impairment can lead to lower speech recognition scores in noise than those with normal cognitive function, with no such difference observed in quiet conditions [28, 29]. This disparity becomes more pronounced during sentence recognition tests [29], suggesting sentence recognition in noise could be a sensitive marker for cognitive decline and potentially reflect underlying WML. However, due to the retrospective design, we lacked data on speech perception in noise or sentence recognition for this study.

Thirdly, a correlation between the Fazekas score and CI performance may not exist. Knopke et al. found a significant correlation only between PVWM score and speech perception in quiet among individuals aged 50–70 [6]. WML are highly prevalent in the elderly, affecting 94–95% of this population [30, 31]. Furthermore, Fazekas et al. showed that 53% of individuals with normal cognitive performance aged 50–70 had cerebral WML, highlighting their nonspecific nature [32]. This suggests that the correlation observed by Knopke et al. may be coincidental. However, this does not rule out the potential correlation between cognition and CI outcomes. The Montreal Cognitive Assessment (MoCA) correlates with speech recognition in noise and explains 35% of the variance post-implantation [10, 33]. Despite this, other studies found no clear link between MoCA scores and CI outcomes [34, 35]. This suggests that while cognition may influence CI outcomes, the mechanism of cerebral WML measured by the Fazekas score may not be specific enough in this study to demonstrate a link.

### MRI abnormalities of the semicircular canals

Several studies have documented the presence of focal sclerosis and narrowing in at least one SCC on Computed tomography (CT) and/or signal loss on T2-weighted MRI in individuals with DFNA9. Our study focused solely on T2-weighted MRI to assess signal loss in the SCCs in DFNA9 subjects, revealing signal loss in at least one SCC in 97.7% of cases. This aligns with Beerten et al. and Ihtijarevic et al., who reported signal loss in at least one SCC in 81.3% and 100% of the DFNA9 subjects, respectively [19, 20]. Additionally, we found the superior SCC to be most frequently affected (48.9%). In contrast, Ihtijarevic et al. reported MRI abnormalities most frequently in the lateral SCC, while Beerten et al. found the posterior SCC most affected [19, 20]. These variations suggest no specific susceptibility of a particular SCC to sclerosis in DFNA9 individuals.

In line with previous research [19, 20], no correlation between the duration or degree of hearing loss and signal loss at the level of the SCCs was detected. Given the fast progressive nature of DFNA9, it can be hypothesized that signal loss in the SCCs will be more frequently observed in subjects with a longer self-reported duration of SNHL or higher degree of hearing loss. The lack of these correlations in our study and previous research is likely due to a selection bias, as suggested by Beerten et al. [36]. Our study focused exclusively on CI recipients, while Beerten et al. and Ihtijarevic et al. examined these correlations in CI candidates and individuals with severe hearing loss, respectively [19, 36]. This indicates that the analyses were conducted at an advanced stage of the disease.

Ihtijarevic et al. found a correlation between hypofunction of caloric responses and MRI abnormalities in at least one SCC [19]. Beerten et al. did not confirm this correlation but found a link between vHIT vestibulocochlear reflex gains in all SCCs separately and the presence of MRI lesions [20]. In our study measuring ENG using rotatory chair testing, 81.1% of subjects with areflexia showed SCC signal loss but no correlation between vHIT results and MRI findings. This lack of correlation might also be influenced by selection bias, as our study included individuals with DFNA9 in a more advanced stage of the disease. Future research evaluating vestibular function and MRI abnormalities at different disease stages of DFNA9 is currently performed in Belgium [37].

### Conclusion and implications for future research

This study demonstrated that most individuals with DFNA9 experience positive outcomes following cochlear implantation. However, the variation in CI outcomes was not related to the degree and localisation of WML. Although it remains possible that this correlation does not exist, our study acknowledges its limitations, emphasizing the necessity for additional research to further assess the potential predictive value of WML on CI outcomes. Subsequent studies should involve prospective designs, incorporating T2/FLAIR MRI conducted at a fixed moment shortly before implantation and assessing outcomes through sentence recognition in noise. Notably, despite the predominantly favourable CI outcomes, less favourable outcomes were observed in a few subjects with a longer duration of SNHL and those who did not use HA pre-implantation. These factors have been previously associated with less favourable CI outcomes.

Furthermore, our study provided additional evidence confirming the high prevalence of focal sclerosis in individuals with DFNA9, with 97.7% of subjects showing signal loss in at least one SCC on T2-weighted MRI. Future research should further dive into exploring the correlation between these findings and the subjects’ vestibular phenotype, aiming to determine whether these observations could aid in clinically diagnosing individuals in a pre-symptomatic stage.

## Electronic supplementary material

Below is the link to the electronic supplementary material.

Supplementary Material 1

Supplementary Material 2

## Data Availability

The datasets used and analyzed during the current study are available from the corresponding author on reasonable request.
