# Supplementary material for: Evaluating cochlear implant outcomes in DFNA9 subjects: a comprehensive study on cerebral white matter lesions and vestibular abnormalities
Source: Eur Arch Otorhinolaryngol. 2024 Sep 13;282(1):183–91. doi: 10.1007/s00405-024-08933-1 (PMC11735485; doi:10.1007/s00405-024-08933-1)
Supplement: Supplementary file 1 — Supplementary Material 1 [file 405_2024_8933_MOESM1_ESM.docx]

**Supplementary table 1. Subject characteristics**

| Subject number | Ear number | Gender | Variant in *COCH** | Self-reported age of onset HL | Implanted ear | Age during implantation | Hearing aid in implanted ear | Vestibular function** | Degree HL pre-implantation*** |
| --- | --- | --- | --- | --- | --- | --- | --- | --- | --- |
| 1 | 1.1 | Female | c.151C>T (p.(Pro51Ser)) | 40 | Right | 55 | Yes | areflexia | Severe |
| 2 | 2.1 | Female | c.151C>T (p.(Pro51Ser)) | 44 | Left | 65 | No | unknown | Severe |
| 3 | 3.1 | male | c.151C>T (p.(Pro51Ser)) | 56 | Right | 63 | Yes | areflexia | Severe |
| 4 | 4.1 | male | unknown | 50 | Right | 79 | Yes | areflexia | Profound |
| 5 | 5.1 | Female | c.151C>T (p.(Pro51Ser)) | 54 | Left | 68 | Yes | areflexia | Profound |
| 6 | 6.1 | Female | c.151C>T (p.(Pro51Ser)) | 47 | Left | 65 | Yes | areflexia | Profound |
| 7 | 7.1 | male | unknown | 41 | Right | 61 | Yes | areflexia | Profound |
| 8 | 8.1 | male | unknown | 58 | Left | 71 | Yes | unknown | Profound |
| 9 | 9.1 | male | unknown | 54 | Left | 69 | Yes | areflexia | Profound |
| 10 | 10.1 | Female | c.151C>T (p.(Pro51Ser)) | 40 | Right | 67 | Yes | areflexia | Profound |
| 11 | 11.1 | Female | c.151C>T (p.(Pro51Ser)) | 50 | Right | 67 | Yes | areflexia | Profound |
| 12 | 12.1 | Female | c.151C>T (p.(Pro51Ser)) | 49 | Right | 54 | No | areflexia | Profound |
| 13 | 13.1 | Female | unknown | 39 | Left | 59 | Yes | unknown | Profound |
|  | 13.2 |  |  |  | Right | 60 | Yes | unknown | Severe |
| 14 | 14.1 | Female | unknown | 40 | Left | 68 | Yes | unknown | Profound |
| 15 | 15.1 | Female | c.151C>T (p.(Pro51Ser)) | 43 | Right | 66 | Yes | areflexia | Profound |
| 16 | 16.1 | male | c.151C>T (p.(Pro51Ser)) | 47 | Right | 66 | No | areflexia | Profound |
| 17 | 17.1 | male | c.151C>T (p.(Pro51Ser)) | 51 | Left | 61 | Yes | areflexia | Profound |
| 18 | 18.1 | Female | unknown | 47 | Right | 57 | Yes | areflexia | Profound |
| 19 | 19.1 | Female | c.151C>T (p.(Pro51Ser)) | 59 | Left | 66 | Yes | areflexia | Profound |
| 20 | 20.1 | Female | c.263G>A (p.(Glyc88Glu)) | 40 | Left | 63 | Yes | areflexia | Profound |
| 21 | 21.1 | Female | c.151C>T (p.(Pro51Ser)) | 42 | Left | 63 | Yes | areflexia | Profound |
| 22 | 22.1 | Female | c.151C>T (p.(Pro51Ser)) | 45 | Right | 59 | Yes | unknown | Profound |
| 23 | 23.1 | Female | unknown | 54 | Left | 70 | Yes | areflexia | Profound |
| 24 | 24.1 | male | unknown | 55 | Right | 65 | Yes | areflexia | Profound |
| 25 | 25.1 | Female | c.151C>T (p.(Pro51Ser)) | 41 | Right | 69 | Yes | areflexia | Profound |
| 26 | 26.1 | male | unknown | 44 | Left | 59 | Yes | areflexia | Severe |
|  | 26.2 |  |  |  | Right | 59 | Yes | areflexia | Profound |
| 27 | 27.1 | Female | c.151C>T (p.(Pro51Ser)) | 40 | Right | 61 | Yes | areflexia | Profound |
| 28 | 28.1 | Female | c.151C>T (p.(Pro51Ser)) | 40 | Left | 56 | Yes | areflexia | Severe |
|  | 28.2 |  |  |  | Right | 56 | Yes | areflexia | Severe |
| 29 | 29.1 | male | c.151C>T (p.(Pro51Ser)) | 40 | Right | 73 | No | areflexia | Profound |
| 30 | 30.1 | Female | c.151C>T (p.(Pro51Ser)) | 51 | Left | 68 | Yes | hyporeflexia | Profound |
| 31 | 31.1 | male | c.151C>T (p.(Pro51Ser)) | 41 | Right | 55 | Yes | areflexia | Severe |
| 32 | 32.1 | male | unknown | 45 | Right | 70 | Yes | areflexia | Profound |
| 33 | 33.1 | male | unknown | 64 | Left | 73 | Yes | areflexia | Profound |
|  | 33.2 |  |  |  | Right | 75 | Yes | areflexia | Profound |
| 34 | 34.1 | male | unknown | 50 | Left | 67 | No | areflexia | Profound |
| 35 | 35.1 | Female | c.151C>T (p.(Pro51Ser)) | 50 | Right | 73 | Yes | unknown | Profound |
| 36 | 36.1 | Female | c.151C>T (p.(Pro51Ser)) | 40 | Left | 69 | Yes | hyporeflexia | Profound |
| 37 | 37.1 | male | c.151C>T (p.(Pro51Ser)) | 51 | Right | 66 | Yes | areflexia | Profound |
| 38 | 38.1 | Female | unknown | 52 | Right | 69 |  | areflexia | Profound |
| 39 | 39.1 | male | c.151C>T (p.(Pro51Ser)) | 38 | Right | 58 | Yes | areflexia | Profound |
| 40 | 40.1 | male | c.151C>T (p.(Pro51Ser)) | 45 | Left | 60 | No | areflexia | Profound |
| 41 | 41.1 | Female | c.151C>T (p.(Pro51Ser)) | 42 | Left | 62 | Yes | unknown | Profound |
| 42 | 42.1 | Female | c.151C>T (p.(Pro51Ser)) | 45 | Left | 69 | Yes | areflexia | Profound |
| 43 | 43.1 | Female | c.151C>T (p.(Pro51Ser)) | 64 | Left | 75 | Yes | areflexia | Profound |
| 44 | 44.1 | Female | unknown | 52 | Right | 68 | Yes | areflexia | Profound |
| 45 | 45.1 | Female | unknown | 44 | Left | 64 | Yes | areflexia | Profound |

HL indicates hearing loss.
* The variant was unidentified, either because a detailed genetic test report was unavailable (N=9) or because the variant was detected in a sibling (N=6).

** Tested with rotatory chair testing using electronystagmography (ENG) and video head impulse test (vHIT).
*** According to WHO’s grades of hearing impairment.
