# Supplementary material for: Evaluating cochlear implant outcomes in DFNA9 subjects: a comprehensive study on cerebral white matter lesions and vestibular abnormalities
Source: Eur Arch Otorhinolaryngol. 2024 Sep 13;282(1):183–91. doi: 10.1007/s00405-024-08933-1 (PMC11735485; doi:10.1007/s00405-024-08933-1)
Supplement: Supplementary file 2 — Supplementary Material 2 [file 405_2024_8933_MOESM2_ESM.docx]

**Table 1*. The Fazekas scoring system***

| **Periventricular white matter lesions (PVWM)** | |
| --- | --- |
| 0 | Absent |
| 1 | Caps or pencil-thin lining |
| 2 | Smooth halo |
| 3 | Irregular periventricular signal extending into the deep white matter |
| **Deep white matter lesions (DWM)** | |
| 0 | Absent |
| 1 | Punctate foci |
| 2 | Beginning confluence |
| 3 | Large confluent areas |

The Fazekas total score is a sum of the PVWM score and the DWM score.

**Table 2*. Subject characteristics***

| Subject characteristic | | N= 45 subjects (100%) | |
| --- | --- | --- | --- |
| Gender, % female | | 28 | (62.2) |
| Implantation |  |  |  |
| Unilateral  Bilateral simultaneously  Bilateral sequentially | | 40  3  2 | (88.9)  (6.7)  (4.4) |
| Variant in *COCH* | |  |  |
| c.151C>T (p.(Pro51Ser)) | | 29 | (64.4) |
| c.263G>A (p.(Gly88Glu)) | | 1 | (2.2) |
| Unknown* | | 15 | (33.3) |
| Pre-implantation ASA score | |  |  |
| 1  2  3 | | 16  28 6 | (32.0)  (56.0)  (12.0) |
| Cardiovascular risk factors | |  |  |
| None  Hypertension  Diabetes Mellitus  High cholesterol  Smoking  Anticoagulant drug use  History of myocardial infarction  History of stroke | | 26  13  9  2  5  6  3  1 | (57.8)  (28.9)  (20.0)  (4.4)  (11.1)  (13.3)  (6.7)  (2.2) |
| Ear characteristic | | **N= 49 ears (100%)** | |
| Age at implantation | | 65±5.9 y | |
| Self-reported duration of hearing loss prior to implantation | | 18±6.3 y | |
| Degree HL pre-implantation** | |  | |
| Severe (61-80 dB HL)  Profound (>80 dB HL) | | 9  40 | (18.4)  (81.6) |
| Hearing aid in ear to be implanted | | 43 | (87.8) |

ASA indicates American Society of Anaesthesiologists; SD, standard deviation; HL, hearing loss; y, years

* The variant was unidentified, either because a detailed genetic test report was unavailable (N=9) or because the variant was detected in a sibling (N=6).

**According to WHO’s grades of hearing impairment.

**Table 3. Magnetic Resonance Imaging (MRI) and vestibular findings**

| Subject characteristic | N= 45 subjects (100%) | | | |
| --- | --- | --- | --- | --- |
| Age at time of MRI | 64±5.6 y | | | |
| Time between MRI and implantation | 7 m (IQR 4-15) | | | |
| MRI sequence |  | | | |
| T2-weighted  FLAIR  T2/FLAIR | 41  1  3 | | | (91.1)  (2.2)  (6.7) |
| Fazekas score |  | | | |
| PVWM  DWM  Fazekas | 1 (IQR 1-2)  1 (IQR 0-1)  2 (IQR 1-3) | | | |
| Additional brain abnormalities |  | | | |
| Previous lacunar infarction  Cerebellar infarction  Cerebellar and lacunar infarction  Infarct left frontal lobe  Tissue loss left frontal lobe  Tissue loss left parietal lobe | 4  1  2  1  1  1 | | | (8.8)  (2.2)  (4.4)  (2.2)  (2.2)  (2.2) |
| SCC characteristics | | **N= 90 ears (100%)** | | |
| Signal loss SCC | |  | | |
| Any canal  Superior canal  Lateral canal  Posterior canal  Missing* | | 70  44  32  31  2 | (77.8)  (48.9)  (35.6)  (34.4)  (2.2) | |
| ENG | |  |  | |
| Areflexia  Not performed | | 68  22 | (75.6)  (24.2) | |
| vHIT | |  |  | |
| Areflexia superior canal  Areflexia lateral canal  Areflexia posterior canal  Not performed | | 31  31  32  58 | (34.4)  (34.4)  (35.6)  (64.4) | |

MRI indicates magnetic resonance imaging; FLAIR, fluid-attenuated inversion recovery; PVWM, periventricular with matter; DWM, deep white matter; SCC, semicircular canal; ENG, electronystagmography; vHIT, video head impulse test; y, years; m, months; IQR, interquartile ranges.

* Signal loss at the level of the SSC was not assessable at the MRI scan of one subject.

**Table 4. Multivariable regression *analysis with the Phoneme scores at 65 dB SPL in quiet as dependent variable.***

| **Variable** | **Unstandardized B** | **Standardized coefficients Beta** | **Standard Error** | **p** |
| --- | --- | --- | --- | --- |
| Constant | 64.069 |  | 22.754 | 0.007 |
| Fazekas | -0.624 | -0.071 | 1.573 | 0.694 |
| Additional brain abnormalities on MRI | 3.1644 | 0.104 | 4.841 | 0.517 |
| Age during implantation | 0.173 | 0.082 | 0.382 | 0.653 |
| Self-reported duration of HL | 0.351 | 0.178 | 0.331 | 0.295 |
| Hearing aid in ear to be implanted | 4.964 | 0.133 | 5.653 | 0.385 |
| Degree of SNHL pre-implantation | -0.041 | -0.047 | 0.143 | 0.776 |

P indicates significancy. R^2^=0.073, p=0.768

**Figure 1.** ***Cochlear implant outcomes***


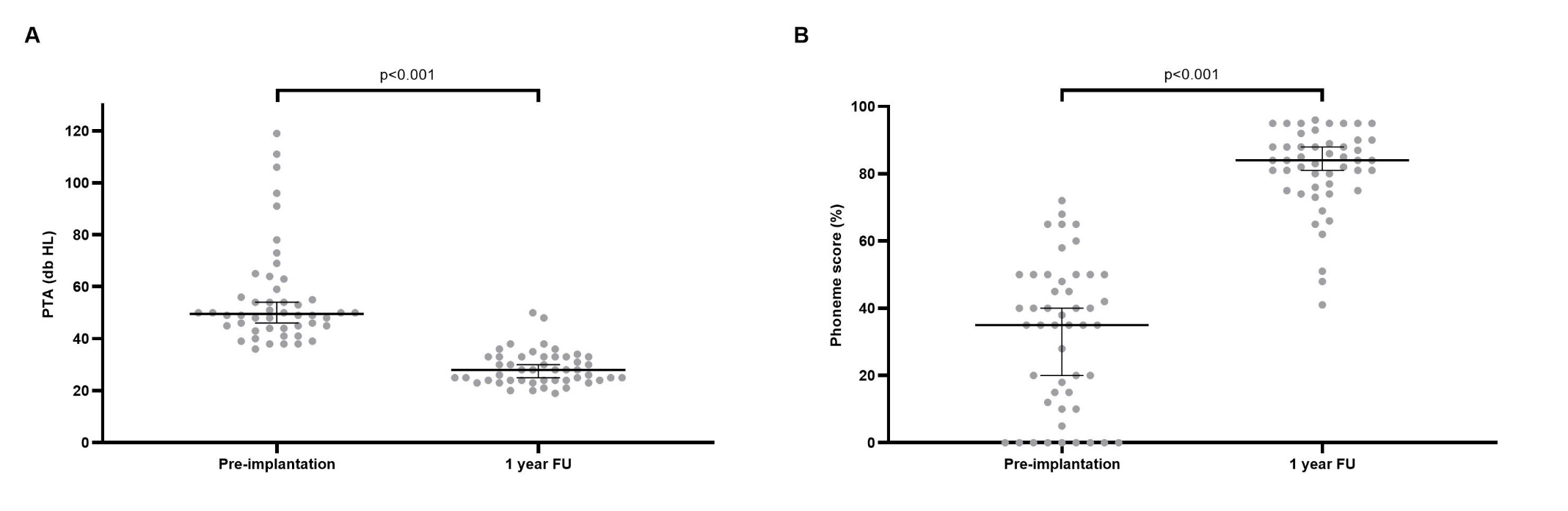


PTA indicates pure tone average; FU, follow-up. Pre-implantation scores represent the best-aided PTA_0,5-4kHz_ and phoneme scores.
**A**. Individual data with median and inter quartile ranges (IQR) of PTA_0,5-4kHz_ scores of each ear. **B.** Boxplot of phoneme scores at 65 dB SPL in quiet of each ear. Pre-implantation aided phoneme scores were not available in all subjects.

**Figure 2.** ***Fazekas score***


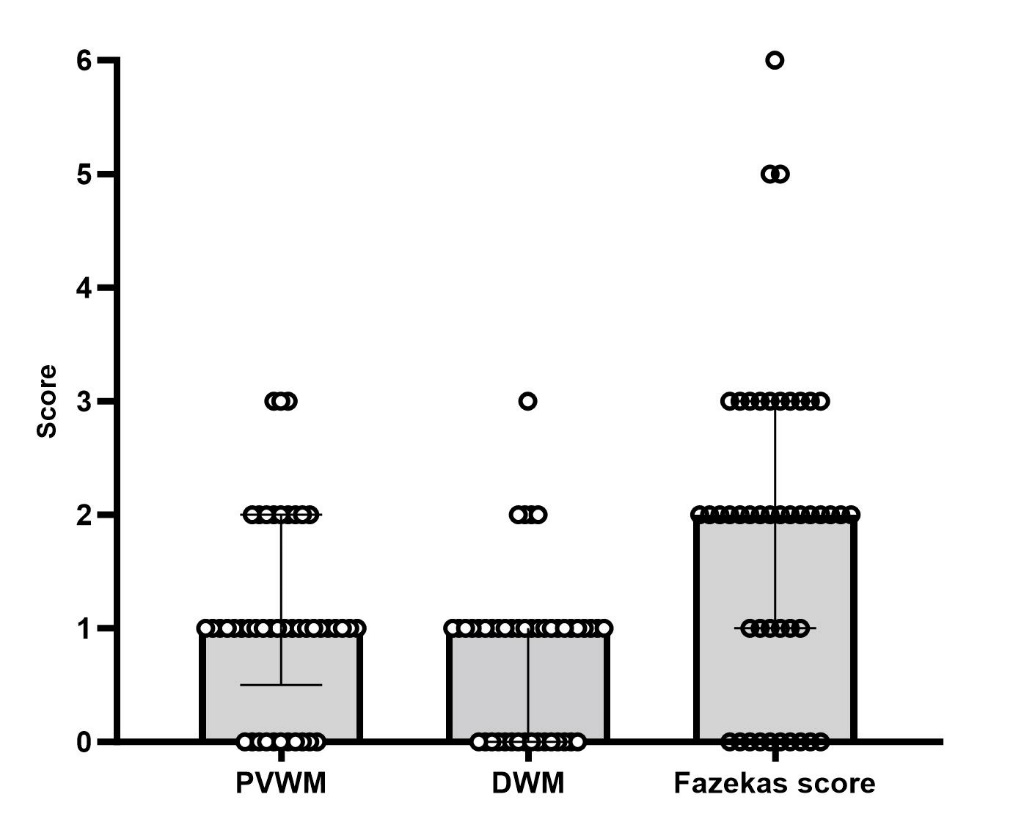


DWM indicates deep white matter; PVWM, periventricular white matter.
Boxplots representing the median scores with interquartile ranges (IQR). The DWM and PVWM score range from zero to three. The Fazekas score is the sum of the PVWM and DWM score and ranges from zero to six. The interpretation of the score is detailed in table 1.

**Supplementary table 1. Subject characteristics**

| Subject number | Ear number | Gender | Variant in *COCH** | Self-reported age of onset HL | Implanted ear | Age during implantation | Hearing aid in implanted ear | Vestibular function** | Degree HL pre-implantation*** |
| --- | --- | --- | --- | --- | --- | --- | --- | --- | --- |
| 1 | 1.1 | Female | c.151C>T (p.(Pro51Ser)) | 40 | Right | 55 | Yes | areflexia | Severe |
| 2 | 2.1 | Female | c.151C>T (p.(Pro51Ser)) | 44 | Left | 65 | No | unknown | Severe |
| 3 | 3.1 | male | c.151C>T (p.(Pro51Ser)) | 56 | Right | 63 | Yes | areflexia | Severe |
| 4 | 4.1 | male | unknown | 50 | Right | 79 | Yes | areflexia | Profound |
| 5 | 5.1 | Female | c.151C>T (p.(Pro51Ser)) | 54 | Left | 68 | Yes | areflexia | Profound |
| 6 | 6.1 | Female | c.151C>T (p.(Pro51Ser)) | 47 | Left | 65 | Yes | areflexia | Profound |
| 7 | 7.1 | male | unknown | 41 | Right | 61 | Yes | areflexia | Profound |
| 8 | 8.1 | male | unknown | 58 | Left | 71 | Yes | unknown | Profound |
| 9 | 9.1 | male | unknown | 54 | Left | 69 | Yes | areflexia | Profound |
| 10 | 10.1 | Female | c.151C>T (p.(Pro51Ser)) | 40 | Right | 67 | Yes | areflexia | Profound |
| 11 | 11.1 | Female | c.151C>T (p.(Pro51Ser)) | 50 | Right | 67 | Yes | areflexia | Profound |
| 12 | 12.1 | Female | c.151C>T (p.(Pro51Ser)) | 49 | Right | 54 | No | areflexia | Profound |
| 13 | 13.1 | Female | unknown | 39 | Left | 59 | Yes | unknown | Profound |
|  | 13.2 |  |  |  | Right | 60 | Yes | unknown | Severe |
| 14 | 14.1 | Female | unknown | 40 | Left | 68 | Yes | unknown | Profound |
| 15 | 15.1 | Female | c.151C>T (p.(Pro51Ser)) | 43 | Right | 66 | Yes | areflexia | Profound |
| 16 | 16.1 | male | c.151C>T (p.(Pro51Ser)) | 47 | Right | 66 | No | areflexia | Profound |
| 17 | 17.1 | male | c.151C>T (p.(Pro51Ser)) | 51 | Left | 61 | Yes | areflexia | Profound |
| 18 | 18.1 | Female | unknown | 47 | Right | 57 | Yes | areflexia | Profound |
| 19 | 19.1 | Female | c.151C>T (p.(Pro51Ser)) | 59 | Left | 66 | Yes | areflexia | Profound |
| 20 | 20.1 | Female | c.263G>A (p.(Glyc88Glu)) | 40 | Left | 63 | Yes | areflexia | Profound |
| 21 | 21.1 | Female | c.151C>T (p.(Pro51Ser)) | 42 | Left | 63 | Yes | areflexia | Profound |
| 22 | 22.1 | Female | c.151C>T (p.(Pro51Ser)) | 45 | Right | 59 | Yes | unknown | Profound |
| 23 | 23.1 | Female | unknown | 54 | Left | 70 | Yes | areflexia | Profound |
| 24 | 24.1 | male | unknown | 55 | Right | 65 | Yes | areflexia | Profound |
| 25 | 25.1 | Female | c.151C>T (p.(Pro51Ser)) | 41 | Right | 69 | Yes | areflexia | Profound |
| 26 | 26.1 | male | unknown | 44 | Left | 59 | Yes | areflexia | Severe |
|  | 26.2 |  |  |  | Right | 59 | Yes | areflexia | Profound |
| 27 | 27.1 | Female | c.151C>T (p.(Pro51Ser)) | 40 | Right | 61 | Yes | areflexia | Profound |
| 28 | 28.1 | Female | c.151C>T (p.(Pro51Ser)) | 40 | Left | 56 | Yes | areflexia | Severe |
|  | 28.2 |  |  |  | Right | 56 | Yes | areflexia | Severe |
| 29 | 29.1 | male | c.151C>T (p.(Pro51Ser)) | 40 | Right | 73 | No | areflexia | Profound |
| 30 | 30.1 | Female | c.151C>T (p.(Pro51Ser)) | 51 | Left | 68 | Yes | hyporeflexia | Profound |
| 31 | 31.1 | male | c.151C>T (p.(Pro51Ser)) | 41 | Right | 55 | Yes | areflexia | Severe |
| 32 | 32.1 | male | unknown | 45 | Right | 70 | Yes | areflexia | Profound |
| 33 | 33.1 | male | unknown | 64 | Left | 73 | Yes | areflexia | Profound |
|  | 33.2 |  |  |  | Right | 75 | Yes | areflexia | Profound |
| 34 | 34.1 | male | unknown | 50 | Left | 67 | No | areflexia | Profound |
| 35 | 35.1 | Female | c.151C>T (p.(Pro51Ser)) | 50 | Right | 73 | Yes | unknown | Profound |
| 36 | 36.1 | Female | c.151C>T (p.(Pro51Ser)) | 40 | Left | 69 | Yes | hyporeflexia | Profound |
| 37 | 37.1 | male | c.151C>T (p.(Pro51Ser)) | 51 | Right | 66 | Yes | areflexia | Profound |
| 38 | 38.1 | Female | unknown | 52 | Right | 69 |  | areflexia | Profound |
| 39 | 39.1 | male | c.151C>T (p.(Pro51Ser)) | 38 | Right | 58 | Yes | areflexia | Profound |
| 40 | 40.1 | male | c.151C>T (p.(Pro51Ser)) | 45 | Left | 60 | No | areflexia | Profound |
| 41 | 41.1 | Female | c.151C>T (p.(Pro51Ser)) | 42 | Left | 62 | Yes | unknown | Profound |
| 42 | 42.1 | Female | c.151C>T (p.(Pro51Ser)) | 45 | Left | 69 | Yes | areflexia | Profound |
| 43 | 43.1 | Female | c.151C>T (p.(Pro51Ser)) | 64 | Left | 75 | Yes | areflexia | Profound |
| 44 | 44.1 | Female | unknown | 52 | Right | 68 | Yes | areflexia | Profound |
| 45 | 45.1 | Female | unknown | 44 | Left | 64 | Yes | areflexia | Profound |

HL indicates hearing loss.
* The variant was unidentified, either because a detailed genetic test report was unavailable (N=9) or because the variant was detected in a sibling (N=6).

** Tested with rotatory chair testing using electronystagmography (ENG) and video head impulse test (vHIT).
*** According to WHO’s grades of hearing impairment.

**Supplementary table 2. C*orrelation analysis with the Phoneme scores at 65 dB SPL in quiet as dependent variable.***

| **Dependant variable** | **Phoneme score at 65 dB SPL in quiet one-year post-implantation (N=49)** | | | | | |
| --- | --- | --- | --- | --- | --- | --- |
|  | **correlations*** | | **Univariate regression analysis** | | | |
| **Variable** | **r_sp_/Pearson’s r** | **p** | **R^2^** | **DF** | **F** | **p** |
| PVWM | 0.022 | 0.822 | 0.001 | 47 | 0.03 | 0.871 |
| DWM | 0.031 | 0.835 | 0.000 | 47 | 0.00 | 0.972 |
| Fazekas | 0.003 | 0.986 | 0.000 | 47 | 0.01 | 0.909 |
| Additional brain abnormalities on MRI | 0.056 | 0.704 | 0.009 | 47 | 0.457 | 0.505 |
| Age during implantation | 0.065 | 0.656 | 0.011 | 47 | 0.519 | 0.475 |
| Self-reported duration of HL | -0.041 | 0.780 | 0.039 | 47 | 1.924 | 0.172 |
| Hearing aid in ear to be implanted | 0.088 | 0.546 | 0.017 | 47 | 0.820 | 0.370 |
| Degree of SNHL pre-implantation | -0.139 | 0.432 | 0.00 | 47 | 0.001 | 0.982 |

r_sp_ indicates spearman’s correlation coefficient; p, significancy; R^2^, squared R; DF degree of freedom; F, F-test.

* Spearman's correlation was employed to test correlations for categorical variables, while Pearson correlation was used for continuous variables.
